# Unraveling the Phylogenetic Relationships of the Eccoptochilinae, an Enigmatic Array of Ordovician Cheirurid Trilobites

**DOI:** 10.1371/journal.pone.0049115

**Published:** 2012-11-16

**Authors:** I. Wesley Gapp, Curtis R. Congreve, Bruce S. Lieberman

**Affiliations:** 1 Department of Geology, University of Kansas, Lawrence, Kansas, United States of America; 2 Department of Ecology and Evolutionary Biology, University of Kansas, Lawrence, Kansas, United States of America; 3 Biodiversity Institute, University of Kansas, Lawrence, Kansas, United States of America; Institut de Biologia Evolutiva - Universitat Pompeu Fabra, Spain

## Abstract

The Cheiruridae are a diverse group of trilobites and several subfamilies within the clade have been the focus of recent phylogenetic studies. This paper focuses on the relationships of one of those subfamilies, the Ordovician Eccoptochilinae. We analyze sixteen species from six genera within the traditionally defined group, using the pilekiid *Anacheirurus frederici* as an outgroup. To assess the monophyly of the Eccoptochilinae seven sphaerexochine species, *Kawina arnoldi*, *Sphaerexochus arenosus*, *S. atacius, S. latifrons, S. mirus, S. parvus,* and *S. scabridus* were included in the analysis as well. The results of this analysis show that the genus *Eccoptochile* represents a paraphyletic grade and species traditionally assigned to *Parasphaerexochus* and *Skelipyx* plot within *Pseudosphaerexochus*. Also, representative species of Sphaerexochinae plot within the traditionally defined Eccoptochilinae, suggesting Eccoptochilinae itself is paraphyletic. To resolve this, we propose all species of *Pseudosphaerexochus* be placed within Sphaerexochinae and Eccoptochilinae be restricted to a monotypic *Eccoptochile clavigera*.

## Introduction

The Cheiruridae Hawle and Corda 1847 [1] are a diverse trilobite group that first appears in the Early Ordovician and persists into the Devonian. Subfamilies within this group have been the subject of recent phylogenetic studies [2]–[4] and have been useful in studying macroevolutionary patterns associated with the Ordovician mass extinction [3]. Other groups of trilobites that persisted concurrently with the cheirurids, such as the aulacopleurids, have also been useful for phylogenetic analysis and the study of paleobiogeographic patterns [5]–[9].

Lane, [10] proposed the Eccoptochilinae as a group within the Cheiruridae, and this is only one of several subfamilial classifications proposed for the Cheiruridae [11]–[15]. Lane [10] contended that the cheirurids be split into seven subfamilies, noting the wide diversity of form within the Cyrtometopinae that Öpik [12] had used to group 14 different genera. Pärnaste [16] agreed with Lane’s assessment of the Cyrtometopinae, redefining the group based on several apomorphies and removing taxa that represented transitional forms between other groups. The Eccoptochilinae was erected by Lane based on a lack of constriction in the thoracic pleaurae (the character, which he used to remove these species from the Cyrtometopinae) as well as a prominent to effaced pitting along a transverse line across the thoracic segments (which added species from the Areiinae and a new genus, *Skelipyx* Lane, 1971 [10]). This new grouping included *Eccoptochile* Hawle and Corda, 1847 [1], *Placoparina* Whittard, 1940 [17], *Pseudosphaerexochus* Schmidt, 1881 [18], *Skelipyx*, and *Arieaspis* Pribyl and Vanek, 1964 [19].

Lane’s assignment was not created within a phylogenetic framework, however, and others have speculated about the efficacy of the subfamily grouping. Pribyl *et al.* [20] disagreed with Lane’s assessment of the group, arguing that Öpik’s [12] original grouping of the Cyrtometopinae was valid and that Lane should not have synonymized *Stubblefeldia* with *Pseudosphaerexochus*.

Whittington [21], in an attempt to address the evolutionary history of the Cheiruridae, hypothesized a theoretical phylogeny for the group. In it, *Pseudosphaerexochus* was grouped with members of the Sphearexochininae and *Eccoptochile* and *Ariea* are a part of a separate lineage. More recently there have been more analytical attempts to assess phylogeny within the Cheiruridae, evaluating individual subfamilies within the group. Studies of the Acanthoparyphinae, Deiphoninae, Sphaerexochinae [2]–[4] have revealed that much of the earlier understanding of the species relationships did not necessarily involve monophyletic groupings.

The purpose of this study is to resolve the phylogenetic relationships within the Eccoptochilinae, a key cheirurid subfamily needing examination in a phylogenetic framework, to test whether the clade is monophyletic and determine its position in relation to the Sphaerexochininae. Taxa analyzed include species classified by Lane [10] within the Eccoptochilinae. Further, six taxa from the Sphaerexochinae (*Sphaerexochus arenosus, S. atacius, S. latifrons, S.* *mirus, S. parvus, S. scabridus,* and *Kawina arnoldi*) were included to assess the monophyly and evolutionary position of the Eccoptochilinae with relation to the Spharexochinae.

## Materials and Methodology

### Phylogenetic Analysis

Morphological terminology follows Whittington [22]. Material was examined with permission at the University of Kansas Museum of Invertebrate Paleontology (KUMIP), Naturhistoriska Riksmuseet, Stockholm, Sweden (AR) the Yale University Peabody Museum of Natural History (YPM), the Museum of Comparative Zoology, Harvard University (MCZ), the VSEGEI in Saint Petersburg, Russia, and the Paleontological Museum of the University of Oslo, Norway (PMO). All material was either loaned or studied on site.

### Taxa Analyzed

Twenty-four taxa were analyzed in this phylogenetic analysis. *Anacheirurus frederici* Salter, 1864 [23] was used as the outgroup as it has previously been suggested [21] that the early Ordovician Pilekiidae are basal to cheirurid subfamilies such as the Eccoptochilinae. Some taxa were excluded from this analysis due to the unavailability of specimens or photographic material or because the material available was poorly preserved or lacking too many characters necessary for the analysis. These species include *Eccoptochile guillieri*, *E. impedita*, *E*. *mariana*, *E. scrobiculata*, *E. vipera*, *Parasphaerexochus tuberculatus*, *Placoparina quadrata*, *Pseudosphaerexochus approximus*, *P. bulbosus*, *P. dubius*, *P. juvensis*, *P. nullicauda*, *P. ovalis*, *P. parallelus*, *P. pater*, *P. ravni*, and *P. wolkae*. *Eccoptochile tumescens* was treated as *E. scuticauda*, following suggestions by Pribyl and Vanek [24] to synonymize the two species.

### Specific Taxa Analyzed

(Relevant material examined is listed where appropriate. In instances where museum material was not examined, species were coded using photographs from scientific publications.) *Anacheirurus frederici*; *Areia bohemica*; *Placoparina sedgwickii*; *Eccoptochile clavigera*; “*Eccoptochile*” *scuticauda*; “*E*.” *almaldensis*; “*E*.” *perlata*; *Pseudosphaerexochus ekphyma*; *P*. *tectus*; *P*. *densigranulatus* (PMO 9455, 94425, 94434, 100.378, 15.60); *P*. *zapata*; *P*. *octolobatus*; *P*. *laticeps*; *P*. *hemicranium* (VSEGEI 23/11059); *P*. *cancrura*; *P*. *roemeri* (VSEGEI 29/11059, 30/11059, 31/11059); *P*. *conformis* (VSEGEI 26/11059, 27/11059); *Kawina arnoldi*; “*Sphaerexochus” arenosus*; *“S.” atacius*; *S. latifrons; S*. *mirus* (AR 39276, 39477–39482, 39484–39486, 39553 a, b; MCZ 1325, 1328, 196479, 196484, 196498; YPM 6573, 183982 183984, 183998–194000; KUMIP 321539–321541); *“S.” parvus*; *“S.” scabridus*.

### Characters

The characters used in phylogenetic analysis are listed below in appropriate order from anterior to posterior position on the organism. A complete character matrix is given in Table 1. Characters emphasize the dorsal exoskeleton of adult, holaspid stage, as ontogenetic information for most of these species is unavailable. Hypostomal characters were not included in this analysis as this information was absent for most taxa included. Any characters regarding size ranges were analyzed to show they were representative of discrete groupings and not continuous.

1. Anterior boarder (0) straight to weakly curved, (1) strongly curved
2. Anterior cephalic boarder visible in dorsal view (0) present, (1) absent [State 0 is represented in Fig. 1.1–3 and state 1 is represented in Fig. 1.4]
3. Proportion of the cephalon that is glabella (0)<50%, (1)>60%
4. Lateral glabellar margins (dorsal view); (0) parallel, (1) straight, expanding anteriorly, (2) curved [State 0 is represented in Fig. 1.1, state 1 is represented in Fig. 1.2, and state 2 is represented in Fig. 1.4]
5. Genae are (0) flat, (1) strongly tilted ventrally
6. S2 and S3 furrows (0) strongly incised, (1) weakly incised, (2) indistinct or absent
7. Anterior most position of the eye (0) abaxial to S3, (1) abaxial to S2
8. S1 (0) as distinct as S2 and S3, (1) more distinct than S2 and S3
9. S1 (lateral) (0) S-shaped; (1) U-shaped
10. SO (0) middle positioned anterior to rest of furrow, (1) straight [State 0 is represented in Fig. 1.3 and state 1 is represented in Fig. 1.1]
11. SO (0) straight (1) concave posteriorly
12. S1 furrow (0) does not intersect SO, (1) intersects SO
13. Genal spines (0) present, (1) absent
14. Number of thoracic segments (0) 11, (1) 9, (2) 12, (3) 10
15. Pitting on thoracic segments (0) absent, (1) present
16. Number of pygidial paired spines; (0) 3, (1) 4
17. Pygidial pleurae (0) appear to be fused, (1) do not appear to be fused
18. Pygidial convexity (posterior) (0) nearly flat, (1) vaulted
19. Pygidial dimensions (0) width approx. equal to length, (1) width approx. twice length
20. First axial ring width (0) 1.5 times greater than width of interpleural field of first pygidial segment, (1) equal to or less than width of interpleural field of first pygidial segment.
21. Furrow on the proximal end of the first pleural spine (0) visible in dorsal view, (1) not visible in dorsal view
22. Orientation of distal ends of first pygidial spines (0) directed straight back, (1) directed abaxially
23. Second pygidial spine (0) strongly curved medially, (1) weakly curved medially or straight
24. Angle the pygidial lateral axial furrow along axial ring 1 and 2 makes with a sagittal line (0) sharp, (1) shallow
25. Distal pleural tips (0) subtriangular, (1) rounded, (2) flat
26. Distal ends of the inner pleural spines (0) gradually taper, (1) expand distally
27. Pleural spines (0) separate from each other distally, (1) terminate close to each other forming pygidial shield [State 0 is represented in Fig. 1.4 and state 1 is represented in Fig. 1.1]
28. Last pleural spines terminate (0) posterior to the second to last pleural spines, (1) anterior to middle pleural spines
29. Terminal axial piece (0) present, (1) absent [State 0 is represented in Fig. 1.3 and state 1 is represented in Fig. 1.4]
30. Last axial ring (0) partially fused, (1) ring not fused, (2) fused completely to terminal axial piece forming a notched shape anteriorly, (3) terminal axial piece absent
31. Lateral edges of terminal axial piece (1) strongly curved, (0) straight sided, (2) absent
32. Terminal axial piece (0) small (sagittal length is equal to or less than the sagittal length of first axial ring), (1) large (sagittal length is equal to or greater than twice the sagittal length of first axial ring), (2) absent
33. Terminal axial piece (sag); (0) short (length equal to width), (1) long (length at least twice as long as wide), (2) absent [Characters 32 and 33 represent two distinct characters and are independent from each other. Character 32 addresses relative overall size whereas character 33 focuses on the relative length of the terminal axial piece.]
34. Distal posterior end of the terminal axial piece (0) rounded, (1) pointed, (2) absent

**Table 1:** Character state distributions for taxa used in phylogenetic analysis.

	1	2	3	4	5	6	7	8	9	10	11	12	13	14	15	16	17	18	19	20	21	22	23	24	25	26	27	28	29	30	31	32	33	34
***Anacheirurus frederici***	0	0	0	0	0	0	0	0	0	0	0	0	0	0	0	0	0	0	0	0	0	0	0	0	0	0	0	X	0	0	0	0	0	0
***Areia bohemica***	0	0	0	0	0	0	0	0	0	X	0	0	?	1	1	0	0	0	1	1	0	0	0	1	0	1	0	0	0	1	0	1	1	0
***Placoparina sedgwickii***	1	0	0	0	0	0	0	0	0	0	1	0	0	2	1	0	0	0	1	1	0	0	0	1	0	1	1	1	0	1	1	1	0	1
***“Eccoptochile” scuticauda***	1	0	0	1	0	0	0	1	0	1	0	0	0	3	1	1	0	0	1	1	0	0	0	0	0	1	0	1	1	3	2	2	2	2
***“E.” almaldensis***	1	0	0	1	0	0	0	1	1	X	0	0	1	2	1	0	0	0	1	1	0	0	1	0	1	1	0	0	0	2	1	1	1	1
***“E.” perlata***	1	0	0	1	0	0	0	1	0	0	0	0	1	2	1	0	0	0	1	1	0	0	1	0	0	1	0	X	0	1	1	0	0	1
***E. clavigera***	1	0	0	1	0	0	0	1	0	0	0	0	1	2	1	0	0	0	1	1	0	0	0	0	1	1	X	0	0	2	0	0	0	1
***Kawina arnoldi***	0	1	1	2	1	0	1	0	0	1	0	0	1	2	0	0	0	1	1	0	1	0	1	0	2	1	1	0	0	1	1	1	0	1
***“Sphaerexochus” atacius***	1	1	1	2	1	2	1	1	0	0	0	0	1	?	0	0	0	1	1	0	1	1	1	0	1	1	1	0	0	0	0	0	0	0
***“S.” parvus***	1	1	1	2	1	2	1	1	0	0	0	X	1	?	0	0	0	1	1	0	1	0	1	0	2	1	1	0	0	0	0	0	0	0
***“S.” arenosus***	1	1	1	2	1	1	1	1	0	1	0	0	1	?	0	0	0	1	1	0	1	0	1	0	1	0	0	0	0	2	1	1	0	1
***S. latifrons***	1	1	1	2	1	2	1	1	0	1	0	1	1	3	0	0	0	1	1	0	1	0	1	0	1	1	1	0	0	2	0	1	1	0
***S. scabridus***	1	1	1	2	1	2	0	1	0	1	0	1	1	?	0	0	0	1	1	0	1	0	1	0	1	1	1	0	0	2	0	1	1	0
***S. mirus***	1	1	1	2	1	2	1	1	1	1	0	1	1	3	0	0	0	1	1	0	1	1	1	1	1	1	1	0	0	2	0	1	1	0
***Pseudosphaerexochus ekphyma***	1	1	1	2	1	0	1	1	1	1	1	1	1	?	0	0	0	0	1	0	1	0	0	0	0	1	0	0	0	0	0	0	0	0
***P. tectus***	1	1	1	2	1	0	1	1	1	1	1	1	1	?	0	1	0	0	1	0	1	0	0	0	0	0	0	1	1	3	2	2	2	2
***P. densigranulatus***	1	1	1	2	1	0	1	1	1	1	0	1	1	?	0	1	1	0	1	0	0	0	0	1	1	0	0	0	1	3	2	2	2	2
***P. zapata***	1	1	1	2	1	0	0	1	1	1	0	1	0	?	0	0	0	0	1	0	1	0	1	0	1	0	0	1	1	3	2	2	2	2
***P. octolobatus***	1	1	1	2	1	0	1	1	1	1	1	X	1	0	0	1	0	0	1	0	1	0	0	0	0	1	0	0	1	3	2	2	2	2
***P. laticeps***	1	1	1	2	1	2	1	1	1	0	X	0	1	0	0	1	0	0	1	0	1	0	0	0	0	1	1	0	1	3	2	2	2	2
***P. hemicranium***	1	1	1	2	1	1	1	1	1	0	0	0	1	2	0	1	0	0	1	0	1	0	1	0	1	0	1	0	1	3	2	2	2	2
***P. cancrura***	1	1	1	2	1	1	1	1	0	0	1	0	0	?	1	1	0	0	1	0	0	1	1	0	1	0	0	0	1	3	2	2	2	2
***P. roemeri***	1	1	1	2	1	1	1	1	1	0	1	0	?	?	0	1	1	0	0	0	1	0	1	0	1	0	0	0	1	3	2	2	2	2
***P. conformis***	1	1	1	2	1	1	1	1	0	0	X	0	0	?	0	1	1	0	0	0	0	0	1	0	1	1	0	1	1	3	2	2	2	2

Characters and character states are as listed in the text. Missing data are indicated by “?”. Character numbers are listed at the top of the table. Character states listed as “X” are polymorphic, where “X” = (0&1).

### Methods

The data were analyzed using TNT v1.1 [25]. A traditional search algorithm (TBR) with 10,000 replications, 1 random seed, and 100 trees saved per replication was used to determine the most parsimonious trees for the data matrix. All characters were unweighted and all multistate characters were treated as unordered as there were no obvious criteria for ordering them. To assess tree support, bootstrap and jackknife values were calculated in TNT. Bootstrap and jackknife tests were analyzed using 10,000 replicates and a traditional search (4 characters, 10 percent of the data, were removed during the jackknife test). The matrix data were compiled into Nexus files using Mesquite v.2.75 [26], and FigTree v.1.3.1 [27] was used to generate the tree figures.

**Figure 1:**
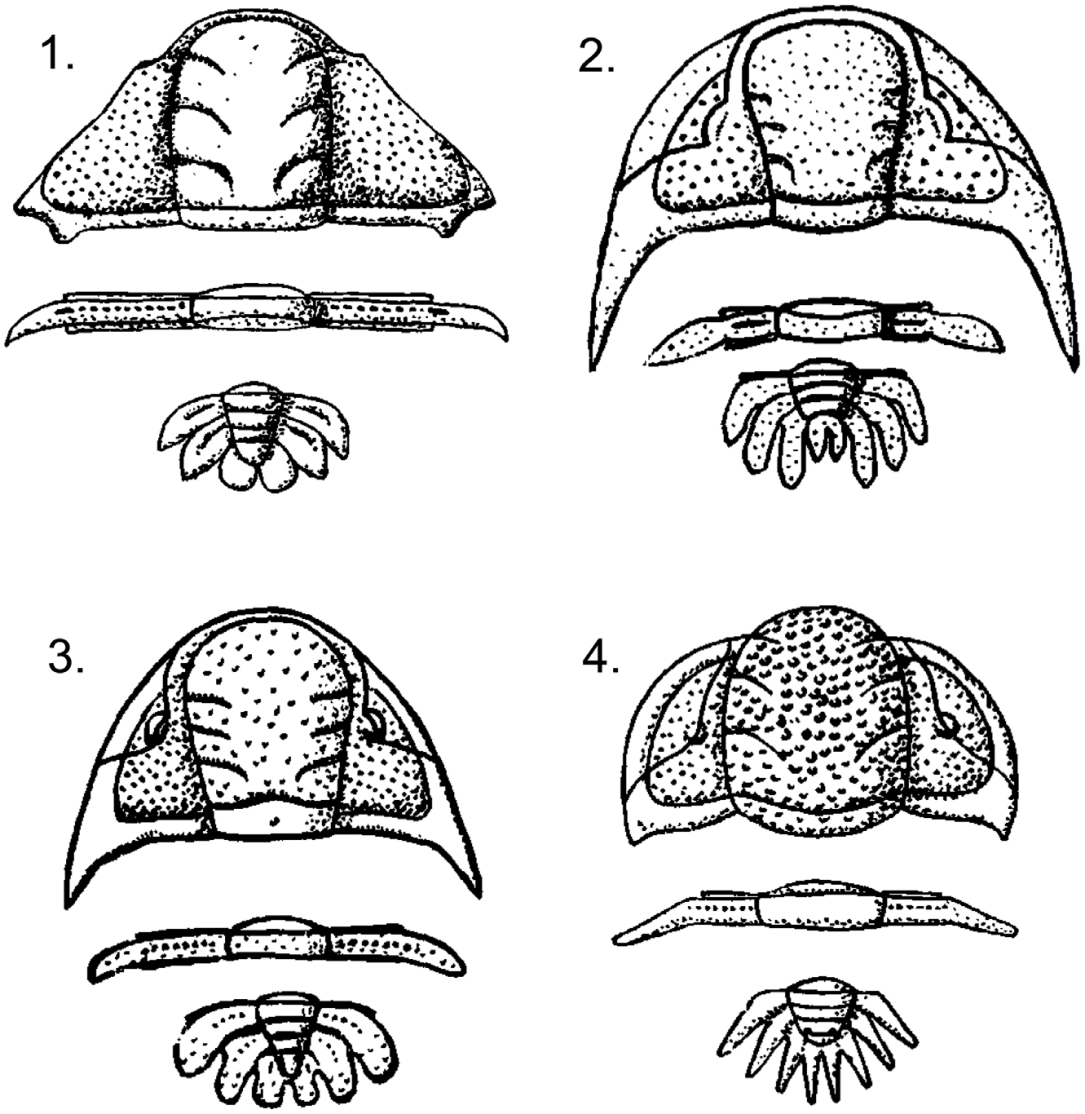
Line drawing of cranidium, thoracic segment, and pygidium of four species traditionally assigned to the Eccoptochilinae. 1, *Placoparina sedgwickii*. 2, “*Eccoptochile” scuticauda*. 3, *Eccoptochile clavigera*. 4, *Pseudosphaerexochus hemicranium*. Modified from Treatise on Invertebrate Paleontology ©1959, courtesy of The Geological Society of America and The University of Kansas.

## Results

Parsimony analysis recovered fourteen most parsimonious trees of length 119 steps with RI values of 0.556, and CI values (when uninformative characters are excluded) of 0.344. A strict consensus of these trees (Fig. 2.1) suggests that taxa traditionally assigned to *Eccoptochile* form a paraphyletic grade basal to *Pseudosphaerexochus* and the Sphaerexochinae. Also, *Parasphaerexochus zapata* and the monotypic *Skelipyx cancrura* fall within *Pseudosphaerexochus*. *Areia* and *Placoparina* plot most basally among ingroup taxa.

**Figure 2:**
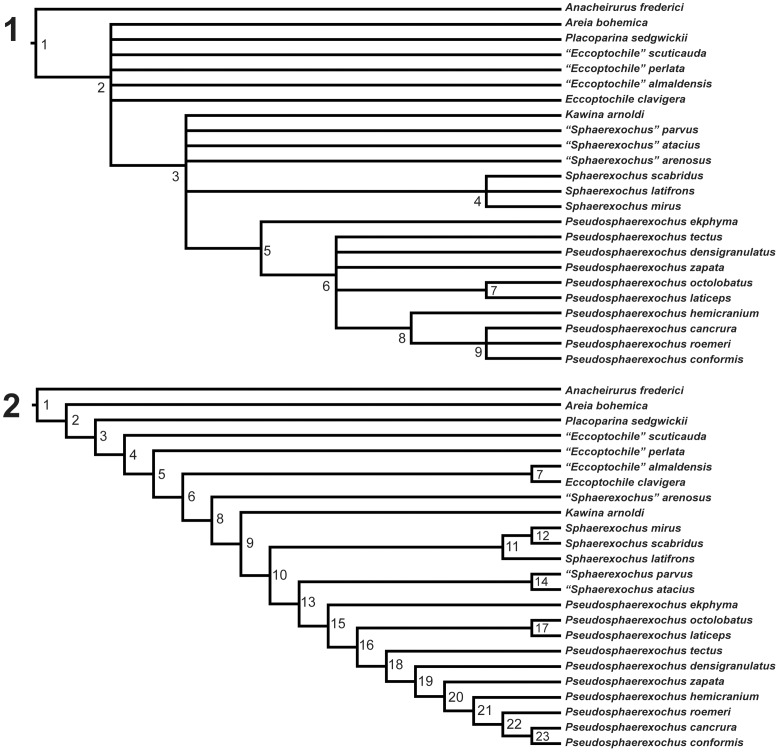
A strict consensus and one of fourteen most parsimonious trees. 1, Results from parsimony analysis showing strict consensus of fourteen most parsimonious trees of length 119 steps. Tree graphics generated using FigTree v.1.3.1 [26] with genera labeled and paraphyletic genus identified using quotations following Wiley [27]. The following nodes of the tree were supported by the following jackknife confidence values (see text for jackknife procedure utilized): Node 2 = 100; Node 3 = 78; Node 4 = 92; Node 5 = 49; Node 6 = 30; Node 7 = 30; Node 8 = 42; Node 9 = 56. The following nodes of the tree were supported by the following bootstrap confidence values (see text for bootstrapping procedure utilized): Node 2 = 100; Node 3 = 29; Node 4 = 57; Node 6 = 7; Node 7 = 2; Node 8 = 8; Node 9 = 18.; *2*, One of fourteen most parsimonious trees of length 119 steps. Most parsimonious character state reconstructions are: *Node* 1∶14[0,1,2]; 15[0,1]; 19[0,1]; 20[0,1]; 24[0,1]; 26[0,1]; 28[0,1]; 30[0,1]; 32[0,1]. *Node 2*∶19(1); 20(1); 26(1); 30(1); 32(1). *Node 3*∶1(1); 14(2); 31(1); 34(1). *Node 4*∶4(1); 8(1). *Node 5*∶13(1); 23(1). *Node 6*∶25(1); 28(0); 30(2). *Node 8*∶2(1); 3(1); 4(2); 5(1); 7(1); 10(1); 15(0); 18(1); 20(0); 21(1). *Node 9*∶27(1). *Node 10*∶6[0,2]; 12[0,1]; 31(0); 34(0). *Node 11*∶6(2); 12(1); 14(3); 33(1). *Node 13*∶16(1); 30(0); 32(0). *Node 14*∶6(2); 10(0). *Node 15*∶9(1); 11(1); 12(1); 18(0); 23(0); 25(0); 27(0). *Node 16*∶29(1); 30(3); 31(2); 32(2); 33(2); 34(2). *Node 17*∶14(0). *Node 18*∶26(0). *Node 19*∶11(0), 25(1). *Node 20*∶13[0,1]; 23(1). *Node 21*∶6(1); 10(0); 12(0). *Node 22*∶11(1); 17[0,1]; 19[0,1]. *Node 23*∶9(0); 13(0); 21(0); Parentheses denote unambiguous optimizations and brackets denote ambiguity.

*“Sphaerexochus” arenosus*, “*S.” atacius, S. latifrons, S. mirus, “S.” parvus, S. scabridus*, and *Kawina arnoldi*, the seven taxa chosen to represent the Sphaerexochinae do not resolve as a monophyletic clade. Based on this analysis, *S. mirus*, *S. latifrons*, and *S. scabridus* group together with the other four taxa creating a grade. The monophyly of this group has been discussed previously by Congreve and Lieberman [4], however these results suggest that the sphaerexochines may represent a paraphyletic grade within the traditionally defined Eccoptochilinae.

## Discussion

Our analysis suggests that the traditional Eccoptochilinae is paraphyletic as the included sphaerexochine species resolved within the other ingroup taxa rather than as an independent lineage. Within the subfamily, the traditionally defined *Eccoptochile* forms a basal paraphyletic grade leading towards the sphaerexochines, and *Parasphaerexochus zapata*, *Skelipyx cancrura*, and various *Pseudosphaerexochus* species. *Pseudosphaerexochus* sits up the tree and is paraphyletic due to the inclusion of *Parasphaerexochus* and *Skelipyx*.

To resolve the issues of paraphyly, *Eccoptochile clavigera* is assigned to a monotypic Eccoptochilinae and *Eccoptochile*. *Areia bohemica*, *Placoparina sedgwickii*, “*E.” scuticauda*, “*E.” perlata*, and “*E.” almaldensis* are removed from Eccoptochilinae and placed within “Eccoptochilinae” using quotation marks to indicate paraphyly *sensu* Wiley [28]. Further, “*E.” scuticauda*, “*E.” perlata*, and *“E.” almaldensis* are removed from *Eccoptochile* and placed within a paraphyletic “*Eccoptochile*.” In this, we are conforming to standard phylogenetic practice by maintaining that all taxanomic definitions should be monophyletic [29].

*Parasphaerexochus zapata* and *Skelipyx cancrura* are herein included within *Pseudosphaerexochus* to make that genus monophyletic. In addition, *Pseudosphaerexochus* is removed from Eccoptochilinae and reassigned to Sphaerexochinae based on the phylogenetic position of the sphaerexochine taxa included in this analysis.

Regarding Lane’s original character diagnosis for the group, lack of constriction in the thoracic pleurae appears to hold true for the Eccoptochilinae and the taxa grading towards it, and we also see some evidence for this among *Pseudosphaerexochus* as demonstrated by *P. octolobatus*, however much of the other taxa within the genus are missing thoracic data to make an assessment of this character’s behavior. Also, it is interesting to note that the pitting along the thorax is present in all ”Eccoptochilinae” and absent in all sphaerexochines (with the exception of *P. cancrura*).

The potential paraphyly within parts of *Sphaerexochus* is to be noted. Congreve and Lieberman [4] had shown that the genus was monophyletic when included in an analysis with species of *Kawina*. By including the Eccoptochilinae with representatives from this group, our analysis suggests that *Pseudosphaerexochus* is a derived sphaerexochine and thus parts of *Sphaerexochus* may not be a monophyletic clade as previously thought. We will not attempt to further revise the taxonomy for this genus as it is not the main focus of this paper and will require further detailed phylogenetic analysis, however it is interesting to note that the subgenus *S. (Sphaerexochus)* does resolve monophyletically, consistent with the results of Congreve and Lieberman [4].

Öpik’s [12] treatment of these groups belonging to a larger Cyrtometopinae appears to be invalid. Our placement of the Sphaerexochinae within Lane’s Eccoptochilinae demonstrates this and provides support for his claims that the Cyrtometopinae had a great range in morphological variation.

Further, our study provides results contradictory to what Whittington [21] had hypothesized for cheirurid relationships. Whittington saw *Eccoptochile* and *Areia* as constituents of a lineage separate from *Kawina*, *Sphaerexochus*, and *Pseudosphaerexochus*. The analysis supports his hypothesis that *Areia* is basal to *Eccoptochile*, however our results suggest Sphaerexochinae derived from these trilobites. Our analysis also disagrees with his suggestion that *Kawina* and *Sphaerexochus* form a separate lineage from *Pseudosphaerexochus*, as our tree indicates that *Pseudosphaerexochus* is a more derived genus that evolves out of *Sphaerexochus*.

### Systematic Paleontology

Family CHEIRURIDAE Hawle and Corda 1847 [1].

Subfamily ECCOPTOCHILINAE Lane 1971 [10].

Genus ECCOPTOCHILE Hawle and Corda 1847 [1].

### Type Species

*Eccoptochile clavigera* (Beyrich 1845) [30].

### Diagnosis

Genae are flat. S2 and S3 are strongly incised and as distinct as S1. S1 furrow is S-shaped and does not penetrate SO. 12 thoracic segments with transverse rows of pitting. The pygidium is shield-like with a small terminal axial piece present.

### Discussion

Because the phylogenetic analysis indicates the traditional *Eccoptochile* is paraphyletic, we redefine it as a monotypic taxon consisting of the type species *E. clavigera*. All other species originally placed within the genus *Eccoptochile* are placed within “*Eccoptochile*” *sensu* Wiley [28].

Subfamily SPHAEREXOCHININAE Öpik 1937 [12].

Genus PSEUDOSPHAEREXOCHUS Schmidt 1881 [18].

### Type Species

*Pseudosphaerexochus hemicranium* (Kutorga 1854) [31].

### Other Species

*P. cancrura* (Salter 1853 [32]), *P. conformis* (Angelin 1854 [33]), *P.* *densigranulatus* Nikolaisen 1965 [34], *P. ekphyma* Lane, 1971 [10], *P.* *laticeps* (Linnarsson 1866) [35], 1991, *P. octolobatus* (McCoy 1849 [36]), *P. roemeri* Schmidt 1881 [18], *P. tectus* Ingham, 1974 [37], *P.* *zapata* (Adrain and Fortey 1997 [38]).

### Diagnosis

Glabella is wide, hides anterior cephalic boarder in dorsal view, with curved lateral margins. Genae are strongly tilted ventrally. The anterior most position of the eye is abaxial of S2. Pitting on the thoracic segment is absent and the first axial ring of the pygidium is wide. The terminal axial piece is absent.

### Discussion

To create a monophyletic genus, *Parasphaerexochus zapata* and *Skelipyx cancrura* are subsumed within *Pseudosphaerexochus*. These taxa share many characters with other members of *Pseudosphaerexochus* that support their placement within the genus. These include a U-shaped S1, a flat pygidium, pleural spines that separate from each other distally, and an absent terminal axial piece. Further, *Pseudosphaerexochus* is removed from Eccoptochilinae and placed within Sphaerexochinae.

Lane’s diagnosis for the genus includes an inflated and ovate glabella with small cheeks and three pairs of lateral furrows, the posterior pair being most distinct. These characters are still valid for describing *Pseudosphaerexochus*, however they are also common among *Sphaerexochus* taxa as well. Lane also noted the short rounded terminal axial piece present in *Pseudosphaerexochus*. This analysis shows that this character was lost within the group with the exception of *P. ekphyma*, which plots more basally to the rest of the group and closer to *Sphaerexochus*. Further, Pribyl et al.’s [20] suggestion that there are two lineages within *Pseudosphaerexochus* based on two pygidial morphotypes does not hold true for our results.

In creating the genus *Skelipyx,* Lane distinguished it from *Pseudosphaerexochus* based on its rounder glabella, much of which is vertical or overhangs. We found the steepness of the lateral margins of the glabella to be very similar between the two genera and that degrees of roundness do not appear to be diagnosably distinct. Lane further notes the unique shape of the pygidium with the wide space between the posterior pair of spines. This character is indeed unique to this taxon, however due to its autapomorphic nature it is not included in this phylogenetic analysis. The placement of *Skelipyx* within *Pseudosphaerexochus* is consistent with Pribyl et al. [20] who assumed *Skelipyx* was derived from that genus.

### Evolutionary Implications

It is interesting to note that, save for the one clade of trilobites belonging to the genus *Sphaerexochus*, all of the other species are restricted to the Ordovician. Furthermore, the early Ordovician species of *Kawina* and *Sphaerexochus* represent the only Laurentian forms, with nearly all other species of “Eccoptochilinae” originating in Avalonia, Bohemia, and Baltica. The topology of our analysis suggests that there may have been a dispersal event early on during the Ordovician that gave rise to the split between *Sphaerexochus* and *Pseudosphaerexochus*. In turn, these Laurentian forms would go on to diversify and dramatically expand their ranges during the Late Ordovician mass extinction [4], while all of the other ”Eccoptochilinae” went entirely extinct. It is possible that dispersal to Laurentia may have been an important factor contributing to the group’s survival. A similar pattern of survivability can be found in the homalonotid trilobites during that time period; most old world homalonotid trilobites went extinction but the one clade that dispersed to Laurentia thrived [39].

## References

[1] Hawle I, Corda AJC (1847) Prodom einer Monograhie der böhmischen Trilobiten. Prague.

[2] Adrain JM (1998) Systematics of the Acanthoparyphinae (Trilobita), with species from the Silurian of Arctic Canada. J Paleontol 72: 698–718.

[3] Congreve CR, Lieberman BS (2010) Phylogenetic and Biogeographic Analysis of Deiphonine Trilobites. J Paleontol 84: 128–136.

[4] Congreve CR, Lieberman BS (2011) Phylogenetic and Biogeographic Analysis of Sphaerexochine Trilobites. PLoS ONE 6: e21304. 21738632 10.1371/journal.pone.0021304 PMC3124496

[5] Rustán JJ, Vaccari NE (2010) The Aulacopleurid trilobite *Maurotarion* Alberti, 1961, in the Silurian-Devonian of Argentina: systematic, phylogenetic and paleobiogeographic significance. J Paleontol 84: 1082–1098.

[6] Rustán JJ, Vaccari NE (2012) The trilobite *Maurotarion megacephalum* sp. nov. (Aulacopleuridae) in the Lower Devonian of Argentina: phylogenetic and paleogeographic remarks. Rev Mex Cienc Geol 29: 346–354.

[7] Ebach MC, Ahyong ST (2001) Phylogeny of the trilobite subgenus Acanthopyge (Lobopyge). Cladistics 17.

[8] Ebach MC, Edgecombe GD (1999) The Devonian trilobite Cordania from Australia. J Paleontol 73: 431–436.

[9] Lieberman BS, Karim TS (2011) Tracing the trilobite tree from the root to the tips: a model marriage of fossils and phylogeny. Arthropod Struct Dev 39: 111–123. 10.1016/j.asd.2009.10.004 19854298

[10] Lane PD (1971) British Cheiruridae (Trilobita). London: Palaeontographical Society.

[11] Evitt WR (1951) Some Middle Ordovician trilobites of the families Cheiruridae, Harpidae and Lichidae. J Paleontol 25: 587–616.

[12] Öpik AA (1937) Trilobiten aus Estland. Acta et Commentationes Universitatis Tartuensis (Dorpatensis) A 32: 1–163.

[13] Prantl F, Pribyl A (1948) Classification of some Bohemian Cheiruridae (Trilobita). Sb nar Mus Praze, 3 Geol (Paleont) 1: 1–44.

[14] Raymond PE (1913) Subclass Trilobita, p. 692–729. In C. R. Eastman (ed.), Textbook of Palaeontology 2nd Ed. New York: The MacMillan Company.

[15] Whittington HB, Evitt WR (1954) Silicified Middle Ordovician trilobites. Mem geol Soc Am: 1–137.

[16] Pärnaste H (2003) The Lower Ordovician trilobite *Krettaspis:* the earliest cyrtometopinid (Cheiruridae) from the Arenig of the East Baltic. Spec Pap Palaeontol 70: 241–257.

[17] Whittard WF (1940) The Ordovician trilobite fauna of the Shelve-Corndon district, west Shropshire. Part 1. Agnostidae, Raphiophoridae, Cheiruridae. Ann Mag Nat Hist 5: 153–172.

[18] Schmidt F (1881) Revision der östbaltischen silurichen Trilobiten nebst geonostischer Übersicht des östbaltischen Silurbebiets. Abt. 1. Phacopiden, Cheiruriden und Encrinuriden. Mem Acad Sci St Petersb 30: 1–237.

[19] Pribyl A, Vanek J (1964) O dvou novych cheiruridních trilobitech z cekeho ordoviku. Ibid 133: 161–166.

[20] Pribyl A, Vanek J, Pek I (1985) Phylogeny and taxonomy of family Cheiruridae (Trilobita). Acta Univ. Palacki. Olomuc. Fac. Med. 83: 107–193.

[21] Whittington HB (1966) Phylogeny and distribution of Ordovician trilobites. J Paleontol 40: 696–737.

[22] Whittington HB, Chatterton BDE, Speyer SE, Fortey RA, Owens RM (1997) Part O; Arthropoda 1; Trilobita revised; Volume 1, Introduction, order Agnostida, order Redlichiida. In: Kaesler RL, ed. Treatise on Invertebrate Paleontology. Lawrence, KS: The University of Kansas Press and the Geological Society of America. 530 p.

[23] Salter JW (1864) A monograph of the British trilobites from the Cambrian, Silurian, and Devonian formations. London: Printed for The Palaeontographical Society.

[24] Pribyl A, Vanek J (1984) Observations on some Bohemian and foreign cheirurid trilobites. Paläont Z 58: 119–130.

[25] Goloboff PA, Farris JS, Nixon KC (2008) TNT, a free program for phylogenetic analysis. Cladistics 24: 774–786.

[26] Maddison WP, Maddison DR (2011) Mesquite: a modular system for evolutionary analysis. 2.75 ed. Available: http://mesquiteproject.org. Accessed 2012 June 1.

[27] Rambaut A (2009) FigTree. 1.3.1 ed.

[28] Wiley EO (1979) An annotated Linnean hierarchy, with comments on natural taxa and competing systems. Syst Zool 28: 308–337.

[29] Wiley EO, Lieberman BS (2011) Phylogenetics: Theory and Practice of Phylogenetic Systematics. 2nd ed.

[30] Beyrich E (1845) Ueber einige böhmische Trilobiten. Berlin: G. Reimer.

[31] Kutorga S (1854) Einige Sphaerexochus und Cheirurus aus den silurischen Kalksteinschichten des Gouvernement St. Petersburg. Zapiska Imperatorskago (SPeterburgskogo) Mineralogicheskogo Obshchestva 13: 105–126.

[32] Salter JW (1853) Figures and descriptions illustrative of British organic remains. *Memoirs of the Geological Survey of the United Kingdom*.

[33] Angelin NP (1854) Palaeontologia scandinavica. P. 1, Crustacea formationis transitionis. Lipsiae: T.O. Weigel.

[34] Nikolaisen F (1965) The Middle Ordovician of the Oslo region, Norway. 18. Rare trilobites of the families Olenidae, Harpidae, Ityophoridae and Cheiruridae. Ibid 45: 231–248.

[35] Linnarsson JGO (1866) Om de Siluriska bilbningarna i mellersta Vertergötland. K svenska Vetensk-Akad Handl 8: 161–179.

[36] McCoy F (1849) On the classification of some British fossil Crustacea with notices of new forms in the University collection at Cambridge. Ann and Mag Nat Hist 2: 161–179.

[37] Ingham JK (1974) The Upper Ordovician trilobites from the Cautley and Dent districts of Westmorland and Yorkshire Part 2. London: The Palaeontographical Society.

[38] Adrain JM, Fortey RA (1997) Ordovician trilobites from the Tourmakeady Limestone, western Ireland. Bull Nat Hist Mus Geol ser 53: 79–116.

[39] Congreve CR, Lieberman BS (2008) Phylogenetic and biogeographic analysis of Ordovician homalonotid trilobites. The Open Paleontology Journal 1: 24–32.

